# Effects of Soil Properties on Pb, Cd, and Cu Contents in Tobacco Leaves of Longyan, China, and Their Prediction Models

**DOI:** 10.1155/2023/9216995

**Published:** 2023-12-28

**Authors:** Wei Xi, YuanYe Ping, HaiYang Cai, Qian Tan, Chaoyang Liu, Junru Shen, YaWen Zhang

**Affiliations:** ^1^College of Primary Education, Zhengzhou Normal University, Zhengzhou 450044, China; ^2^Xinjiang Institute of Ecology and Geography, Chinese Academy of Sciences, Urumqi 830011, China; ^3^College of Resources and Environment, Fujian Agriculture and Forestry University, Fuzhou 350002, China; ^4^Guangzhou Lanshen Technology Co., Ltd., Guangzhou 510000, China

## Abstract

Longyan City in Fujian Province is one of China's top-quality tobacco-producing areas and plays an essential role in local economic development. To determine the correlation between heavy metal content in tobacco leaves and soil factors, soil physical and chemical properties and heavy metal contents of lead, cadmium, and copper in tobacco leaves were measured and analyzed by the correlation regression method. The content of lead, cadmium, and copper in soil was determined using hydrochloric acid extraction-AAS and graphite furnace atomic absorption spectrometry. Inductively coupled plasma-mass spectrometry was used to determine heavy metal in tobacco leaf. The findings revealed that the average concentrations of lead, cadmium, and copper in the soil were 12.1, 0.092, and 3.88 mg/kg, respectively. In contrast, the average levels of lead, cadmium, and copper in tobacco leaves were 2.33, 4.89, and 4.35 mg/kg, respectively. The cadmium enrichment coefficient of 54.3 was higher than lead and copper, indicating a greater health risk. Soil pH value was negatively correlated with lead content in tobacco leaf, while potassium and phosphorus nutrient levels were negatively correlated with copper content. In contrast, a positive correlation was established between the presence of organic matter with cadmium content in tobacco leaves. The prediction models of lead, cadmium, and copper in tobacco leaves can be expressed by the regression equation corresponding to each heavy metal as follows: *Y*_Pb_=2.33 − 0.005*∗* *X*_K_+0.007*∗X*_N_  − 0.271*∗X*_pH_+0.065*∗X*_Pb_ (*R*^2^ = 0.787), *Y*_Cd_=1.55+0.012*∗X*_OM_ − 0.014*∗X*_Cu_+34.6*∗X*_Cd_ (*R*^2^ = 0.891), and *Y*_Cu_=4.64 − 0.029*∗X*_P_ − 0.007*∗X*_K_+0.245*∗X*_Cu_ (*R*^2^ = 0.724), respectively. The prediction models above provide an effective predictive tool for assessing heavy metal risk in tobacco leaves using soil properties in the study area.

## 1. Introduction

The unprecedented growth of the human population has resulted in a surge of anthropogenic activities, including industrialization, urbanization, metal mining, and agricultural development, which have led to a substantial accumulation of heavy and toxic metals in the natural environment, posing a grave threat to farmland ecosystems. This has propelled heavy metal pollution to become a pressing and progressively worsening global issue [1, 2]. Of the various types of pollution in farmland ecosystems, water and air pollution are more readily observable, whereas heavy metal pollution in the soil is often concealed and not immediately apparent. It may only become evident through the noticeable impact on human health from consuming crops, including food, vegetables, and fruits. Zheng et al. confirmed that heavy metals could exert health risks to humans due to the consumption of crops [3]. Furthermore, heavy metals are difficult to degrade by microorganisms once they enter the soil and continue accumulating, posing potential and ongoing ecological risks to the soil and crops. The accumulation of heavy metals in the soil beyond a certain threshold level can poison both the soil and the plant system, resulting in soil degradation and reduced crop yield and quality and contaminating surface water and groundwater via runoff and leaching. The potential hazards to human life and health arising from direct contact or through the food chain cannot be overlooked [4–6].

Heavy metal stress in plants can result in micronutrient deficiencies and reduced enzyme availability, especially in cash crops. Additionally, high levels of heavy metals can hinder the absorption and transport of essential minerals, such as calcium and magnesium, leading to growth inhibition of leaves and roots, as well as a reduction in the absorption capacity of key nutrient elements like ferrum, calcium, magnesium, and kalium. The average growth of cash crops is affected and poses a risk to human and animal health if consumed. Therefore, the accumulation of heavy metals in soil and cash crops has attracted considerable attention [7–9].

However, tobacco consumption is an essential way for metals to accumulate in the human body, which has been overlooked. The heavy metals accumulated in tobacco leaves can enter the human body through inhaling the smoke when a cigarette is burned, causing potential harm to human health [10]. Heavy metals, especially lead (Pb), cadmium (Cd), and cuprum (Cu), are highly toxic and can only be tolerated at low concentrations. Excessive levels of these metals can cause various adverse health effects, including depletion of essential nutrients in the body, damage to the esophagus and larynx, impaired psychosocial behavior, weakened immune defenses, reproductive disorders, and cancer [11, 12]. As a nonessential element, Pb mainly causes harm to the human nervous system, hematopoietic system, and kidneys. It can damage the skeletal hematopoietic system and cause anemia, cerebral hypoxia, edema, and abnormal movements and sensations [12]. Cd is highly toxic and can accumulate in vital organs, particularly the kidneys and liver, affecting multiple enzymes. Cd-induced renal damage has been shown to reduce the activity of delta-aminolevulinic acid synthetase, lipoamide and alcohol dehydrogenase, and arylsulfatase. On the other hand, Cd exposure can enhance the activity of delta-aminolevulinic acid dehydratase, pyruvate decarboxylase, and pyruvate dehydrogenase in the human body [12]. Copper is an essential micronutrient that plays a critical role in healthy hormone secretion, nerve conduction, biological electron transfer processes, bone and connective tissue growth, and the synthesis of red blood cells [13]. It is also important for seed production, regulation of water, and resistance to diseases in plants [14]. Thus, research on preventing and controlling heavy metal accumulation in tobacco has become essential to tobacco quality and safety control.

Longyan city is a renowned producer of high-quality tobacco and one of China's three major flue-cured tobacco-producing regions. Previous research mainly focused on soil nutrients, spatiotemporal pH changes, aroma components of tobacco leaves and their critical environmental impact factors, and the influence of soil microorganisms in tobacco fields on tobacco leaf quality. For example, Liu et al. conducted a regional study on the primarily available nutrients in tobacco planting soil in Longyan city [15]. The results showed that the primarily available nutrients in the soil for tobacco planting in this area were moderately variable in alkali-hydrolyzable nitrogen, and both available phosphorus and available potassium were strongly variable; the areas where alkaline hydrolysis nitrogen, available phosphorus, and available potassium in tobacco planting soil were deficient accounted for 25.50%, 2.20%, and 45.60% of the total cultivated land area, respectively. Qian et al. conducted a study on the spatiotemporal variation of soil pH in the Longyan tobacco planting area: soil acidification was effectively controlled, the area unsuitable for planting flue-cured tobacco first increased and then decreased, and the most suitable planting area showed a decreasing trend [16]. Chen et al. researched the distinct aroma components of Longyan tobacco leaves and their critical ecological influencing factors [17]. They gained some new understandings: Longyan tobacco leaves' main characteristic aroma component is beta-damascenone and the main ecological factors affecting aroma components are meteorological factors. Among meteorological factors, the spatial and temporal distribution of precipitation has the most significant influence on the characteristic aroma components of Longyan tobacco leaves. Lai et al. conducted experiments on interplanting garlic's effects on tobacco field soil microorganisms and tobacco leaf quality in the Longyan Tobacco Area [18]. The results showed that interplanting garlic in tobacco fields could significantly reduce the number of bacteria and fungi in the soil of tobacco fields and control the occurrence and damage of tobacco rhizome diseases, thereby reducing the use of chemical pesticides and improving the safety of tobacco leaves.

However, the impact of soil physical and chemical properties, including Pb, Cd, and Cu, on heavy metal content in tobacco leaves in the field and nonlaboratory environments have yet to be systematically analyzed. Those seriously restrict the effective control of heavy metals in tobacco and tobacco products are not conducive to the construction of a healthy tobacco-growing soil environment and the sustainable development of flue-cured tobacco production. Therefore, we chose tobacco in Longyan city as the research object. By analyzing the contents of Pb, Cd, and Cu in tobacco leaves and their correlation with soil environmental factors, we establish a quantifiable relationship between heavy metal content in tobacco leaves and the main soil control factors. Furthermore, the prediction of heavy metal accumulation in plants is a rapidly developing field of research that has been successfully applied in a variety of fields [14]. In this paper, we established quantitative relationships and prediction models by analyzing the contents of Pb, Cd, and Cu in tobacco leaves and their correlation with soil environmental factors, in order to provide a scientific basis for the evaluation and prediction of metal pollution in tobacco production in this area.

## 2. Background of the Study Area

**Figure 1:**
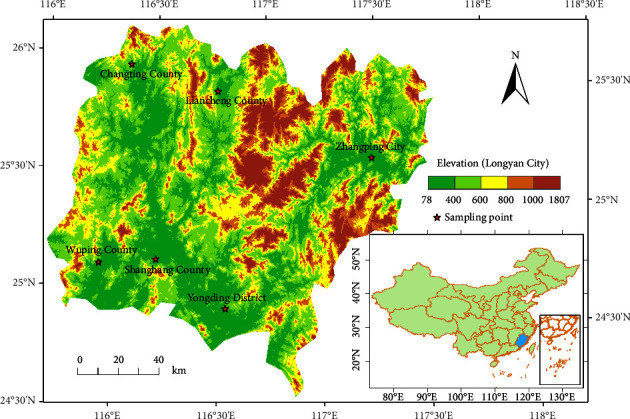
Location map of the study area (Longyan city).

Longyan City is situated in the northern-central region of Fujian Province, China, with coordinates ranging from 24°23′ to 26°02′N and 115°51′ to 117°45′E (refer to Figure 1). The city covers an area of 19,050 km^2^, stretching approximately 192 km from east to west and 182 km from north to south, making up 15.7% of the province's total area. The city is comprised of 14964 km^2^ of mountains, 3101 km^2^ of hills, and 985 km^2^ of plains, respectively. A high elevation in the east and west and a low elevation in the north and south characterize the terrain.

Longyan's tobacco-growing area is mild all year round, with a subtropical marine monsoon climate. The average annual temperature ranges from 18.7°C to 21.0°C, with an average precipitation of 1031 mm to 1369 mm and an average sunshine duration of 1804 to 2060 hours. This favorable climate makes it ideal for growing crops and trees in the subtropical zone, with rice-tobacco rotation being the area's primary method of tobacco planting.

Longyan city's tobacco industry, which began in the late 1970s and early 1980s, is one of its six key industries and plays a significant role in agriculture production. According to the Tobacco Research Institute of the Ministry of Light Industry's ecological grade zoning standards for flue-cured tobacco in tobacco planting, the Longyan area is rated as one of the three most suitable ecological areas for flue-cured tobacco production in China.

## 3. Samples and Methods

### 3.1. Sample Collection

The sampling locations for flue-cured tobacco in Longyan city were Shanghang County, Changting County, Yongding District, Liancheng County, Wuping County, and Zhangping City, as shown in Figure 1. A total of 421 soil samples were collected at a depth of 20 cm below the ground using the plum spot sampling method, which entails sampling five points in a unit area—covering the east, west, south, north, and center. Detailed sampling procedures and cases are available in [19]. Another 421 tobacco leaf samples were collected at the same locations as the above 421 soil samples. The soil samples were air-dried, treated to remove impurities, ground, sieved, mixed, and stored in plastic bottles. The tobacco leaf samples were collected from the same sites as the soil samples and from the tobacco plants' middle leaves. The leaves were washed with ionized water, killed in an oven at 100–150°C for 5–10 minutes, dried at 70–80°C to a constant weight, ground, screened (0.149 mm), marked, and stored in sealed plastic bags for later analysis.

### 3.2. Analysis Method

The soil samples were digested using the hydrochloric acid-nitric acid-Perchloric acid method and filtered. Some of them were extracted by diethylenetriaminepentaacetic acid (DTPA) extraction method, and graphite furnace atomic absorption spectrometry was used to determine the content of Pb and Cd in soil. The other part used 0.1 mol/L HCl extraction method and atomic absorption spectrometry (AAS) method to determine Cu content in soil. Detailed steps can be found in the literature [20, 21].

The content determination of Pb, Cd, and Cu in tobacco leaves adopts inductively coupled plasma optical emission spectrometer (ICP-OES). The instrument model is PerkinElmer-Optima 8300 from the United States. The test procedure can be summarized as follows: Firstly, 0.2000 g of tobacco leaf sample was weighed, 5 mL of nitric acid and 1 mL of perchloric acid were added, and a small funnel was added to the mouth of the bottle to stand overnight. The next day after the sample digestion to white smoke, we continue to add an appropriate amount of ultrapure water to drive acid and then continue to dissolve it. When the sample was white again and 1 mL remained, we put the sample into a volumetric bottle for constant volume and dilution. Finally, the content of Pb, Cd, and Cu in tobacco leaf can be measured with ICP-OES instrument. Detailed steps can be found in the literature [22, 23].

Soil physical and chemical properties were determined as follows: The pH of soil samples was analyzed by the potentiometric method. The pH meter to measure acidity is P901 produced by Shanghai Yuke Instrument and Equipment Co., LTD. Soil pH measurements have a ratio of soil to water of 1 : 5, with soil in grams and water in milliliters. Soil organic matter content was determined by Elementer Vario MAX CN element analyzer. Alkali-hydrolyzed nitrogen content was determined by alkali-hydrolyzed diffusion method. After extracting from sodium bicarbonate, the content of available phosphorus was determined by spectrophotometer. The available potassium was extracted with 1 mol/L ammonium acetate and determined by flame photometer. The analysis of soil electrical conductivity (EC) was performed with the DDSJ-308F conductivity meter of Shanghai Yidi Electrical Scientific Instrument Co., LTD. Total organic carbon (TOC) analysis of soil was conducted by the TOc-L-Cph-SSM5000A analyzer of Shimazu Company. Detailed steps can be found in the literature [24–26].

### 3.3. Statistical Analysis

SPSS (20.0) software was used to perform Pearson correlation and multiple linear regression analyses. ArcGIS 10.8 and Office 2019 software were used to analyze Pb, Cd, and Cu content characteristics in the soil.

## 4. Results and Analysis

### 4.1. Physical and Chemical Properties for Soil

Studies have shown that the optimal growth of root systems and nutrient absorption in plants require suitable soil conditions, including appropriate pH, effective phosphorus, organic matter, alkaline nitrogen, and quick-acting potassium [27, 28]. The soil analysis in the Longyan tobacco area revealed that the soil is typical of the acidic southern region of China (Table 1). It has a relatively high organic matter content, and the three fast-acting nutrients N, P, and K are also in suitable ranges. The soil pH range was found to be 3.37–8.64, with an average value of 5.81 and a low coefficient of variation (8.1%). 95% of the soil falls within the 5.5–6.5 pH range, indicating that the soil is mainly weakly acidic. This is beneficial for tobacco growth, typical of southern China soils. The organic matter, alkaline nitrogen, effective phosphorus, and quick-acting potassium content in the area showed a wide range of values but with overall high averages of 29.1 g/kg, 99.5 mg/kg, 37.4 mg/kg, and 26.1 mg/kg, respectively. The variation coefficients, excluding potassium (7.91%), are low. The mean values of EC and TOC were 0.587 (mS/cm) and 31.5 (g/kg), respectively, which were within the suitable range for tobacco growth and showed little change.

### 4.2. Heavy Metals in Soil

The soil in the study area was analyzed for the presence of Pb, Cd, and Cu heavy metals, as shown in Table 2. The results revealed that the average Pb content was 12.1 mg/kg, with a low coefficient of variation with 5.79%. The minimum Pb content was 1.39 mg/kg, while the maximum value was 49.9 mg/kg. Most of the samples had Pb content lower than the second-level index of agricultural soil heavy metal pollution in Fujian Province (28.0 mg/kg), with an overstandard rate of only 0.951%.

The Cd content was low, with an average value of 0.092 mg/kg, a minimum of 0.003 mg/kg, and a maximum of 0.950 mg/kg. All the samples were lower than the second-level index of agricultural soil heavy metal pollution in Fujian Province (0.150 mg/kg), with an overstandard rate of 12.1%.

The Cu content was relatively higher, with an average value of 3.88 mg/kg, a minimum of 0.031 mg/kg, and a maximum of 19.1 mg/kg. Although most of the samples had Cu content lower than the second-level index of agricultural soil heavy metal pollution in Fujian Province (15.0 mg/kg), there was an overstandard rate of 1.12%.

In conclusion, the heavy metal content in the soil of the study area is generally low and within the acceptable range, which should not cause significant harm to the growth of tobacco.

### 4.3. Heavy Metals in Tobacco Leaves

Table 3 reveals the levels of Pb, Cd, and Cu in tobacco leaves in the study area. The range of Pb in tobacco leaves is 0.430–7.08 mg/kg, with an average of 2.33 mg/kg. The range of Cd in tobacco leaves is 0.221–13.2 mg/kg, with an average of 4.89 mg/kg. The range of Cu in tobacco leaves is 1.23–9.15 mg/kg, with an average of 4.35 mg/kg. The coefficients of variation for Cd, Pb, and Cu are relatively low.

The enrichment coefficient of tobacco leaves refers to the ratio of heavy metals in the tobacco leaves to the heavy metal content in the soil. The average enrichment coefficient of Cd in tobacco leaves from the Longyan tobacco area is 54.3, while the enrichment coefficient of Pb is 19.3, and the enrichment coefficient of Cu is 1.07. This indicates that tobacco leaves are high accumulators of Cd and medium accumulators of Pb but have a weak ability to absorb heavy metals from the soil. Corresponding to this, although tobacco is a low accumulator of Cu, its absorption capacity for soil heavy metals is strong.

### 4.4. Correlation

Table 4 displays the correlation between the heavy metal content of tobacco leaves and the effective state content of heavy soil metals. The results indicate a significant positive correlation between the Pb content of tobacco leaves and soil Pb, with a correlation coefficient of 0.671 (*P* < 0.001). Additionally, the Pb content of tobacco leaves also shows positive correlations with soil pH (−0.301), N (0.331), and K (0.164).

The Cd content of tobacco leaves is also positively correlated with soil Cd, with a correlation coefficient of 0.877 (*P* < 0.001).

The Cu content of tobacco leaves has a significant positive correlation with soil Cu, but there is also a significant negative correlation between Cu content in tobacco leaves and soil K (−0.289) and P (−0.538).

### 4.5. Prediction Models

**Figure 2:**
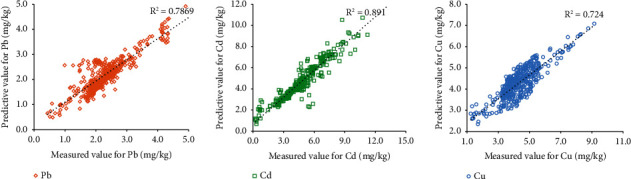
Scatter plot of predicted versus measured amount of Pb, Cd, and Cu, respectively.

The experience models established through gradual linear regression (Figure 2) shows that soil heavy metal content and physical and chemical properties have the greatest impact on the content of tobacco leaf Pb, Cd, and Cu. The results indicate that soil pH, effective N, effective K, and effective Pb have the greatest impact on Pb content in tobacco leaves, while soil K and P content have a greater impact on Cu content in tobacco leaves.

According to the regression equation, the fitting of the predictive value for Cd content in tobacco leaves is the best, while the degree of fitting for Pb and Cu content is relatively poor (as shown in Table 5).

## 5. Discussion

### 5.1. Association of Heavy Metals in Tobacco Leaves with Soil

The ways heavy metals enter tobacco can be divided into two categories: those that enter through the leaf surface from the atmosphere and those that enter through the tobacco roots from the soil. Christensen believed that soil's physical and chemical properties were the main factors affecting the absorption of heavy metals by plants [8]. Notably, the levels of Pb, Cu, and Cd in tobacco leaves exhibit significant variations among different tobacco-growing regions. This observation is consistent with previous findings highlighting the soil as the main reservoir of heavy metals in tobacco. Indeed, discernible disparities are evident in the Pb, Cu, and Cd concentrations across diverse tobacco production areas [29]. Zhang et al. studied the heavy metal content in tobacco leaves from 10 provinces and regions in China [30]. She found that, on average, the content of Pb and Cd in tobacco leaves from northern China was lower than that from southern China, except for Cu, which showed no significant difference. Our study area was in the southern region, and we found that, compared to other regions, the average content of Cd and Cu in tobacco leaves was higher, and that of Pb was medium. The average Pb content was 2.33 mg/kg, Cd was 4.89 mg/kg, and Cu was 4.35 mg/kg.

In the soil-plant system, the plant's Pb, Cd, and Cu accumulation is influenced by many factors, such as soil pH, organic matter content, and soil metal concentration [31, 32]. Soil pH is a crucial factor affecting the transfer of heavy metals to plants [33–35]. Soil pH can affect the Pb, Cd, and Cu levels in tobacco leaves by affecting the availability of these heavy metals in the soil solution. Acidic soils with low pH levels tend to have higher levels of heavy metal ions, making them more available for uptake by the tobacco roots. Conversely, bare soils with high pH levels tend to have lower levels of heavy metal ions, leading to lower accumulation in the tobacco leaves. The soil pH can also influence other factors, such as organic matter content, which can further impact the availability and accumulation of heavy metals in tobacco plants. Studies have found that pH mainly affects the adsorption capacity of Pb and Cd in soil. In the pH 4.00–7.70 range, the adsorption capacity of Pb and Cd increases three times with each unit rising in pH value, which reduces the migration capacity of Pb and Cd to plant rhizosphere [36]. Golia et al. further stated that soil pH was critical for heavy metal migration in plant-soil systems [37]. For instance, in the carrot-soil pot experiment, lettuce-soil experiment, and spinach-soil experiment conducted by [22], pH emerged as the primary soil factor influencing Cd absorption, with soil organic matter following closely. In our study, 95% of the soil falls within the 5.50–6.50 pH range with an average value of 5.81. The pH had a more noticeable effect on Pb content in tobacco leaves, showing a negative correlation, while Cd and Cu showed no obvious correlation. This indicates that moderately acidic soil is more conducive to tobacco plants' absorption and accumulation of Pb. Shao et al. also confirmed this by studying the effects of biochar on the growth of flue-cured tobacco, the properties of rhizosphere soil, and the content of heavy metals in leaves [38]. Organic matter content and soil nutrient components such as nitrogen, phosphorus, and potassium can also affect the accumulation of heavy metals in tobacco leaves [27, 28]. Generally, the higher the organic matter content, the lower the accumulation of heavy metal content in plants [39–41]. The main component of organic matter is humus. Although the complexes formed by humic acid and humin with heavy metals are not readily soluble, fulvic acid is more soluble than those of heavy metals. Heavy metal complexes can increase their bioavailability by increasing the concentration of heavy metal ions in soil solution through the dissolution of the mineral surface. With the increase of soluble heavy metal complexes, Pb, Cd, and Cu in soil are more easily transferred to plants. Our results showed that the content of Cd in tobacco leaves in this region was closely related to soil organic matter and showed a positive correlation. These are consistent with previous research. For example, Regassa and Chandravanshi analyzed the heavy metal content of tobacco leaves from two different regions of Ethiopia (Billate and Shewa Robit) [42]. They found that the average Cd content was 1.25, positively correlated with soil organic matter, while for Pb and Cu elements, no such links were found. Liu et al. found that Cd, Pb, and organic matter had a very significant positive correlation after sampling and analyzing the tobacco-growing areas in Southwest China [43]. The contents of nitrogen, phosphorus, and potassium in soil affected the contents of Pb, Cd, and Cu in tobacco leaves by affecting plant growth and nutrient absorption. High levels of these essential nutrients can stimulate plant growth and increase the absorption of heavy metals, leading to more accumulation in tobacco leaves. Conversely, low levels of these nutrients can limit plant growth and reduce the absorption of heavy metals, leading to less accumulation in tobacco leaves. In addition, unbalanced levels of nitrogen, phosphorus, and potassium can alter the balance of other elements in the soil and affect the availability and absorption of heavy metals. Zhaoxiang et al. conducted a study using pot experiments to understand the relationship between heavy metal content in tobacco leaves and soil properties in Changsha, China [44]. It was found that Pb and Cu elements in tobacco leaves in this area were easily affected by nitrogen, phosphorus, and potassium in the soil, showing a correlation. Therefore, the content of nitrogen, phosphorus, and potassium in soil plays an important role in the determination of heavy metal content in tobacco leaves. Our results showed that nitrogen, phosphorus, and potassium had obvious effects on the content of Pb and Cu in tobacco leaves. This suggests that appropriate nitrogen, phosphorus, and potassium levels in the region are a guarantee to ensure the safe production of tobacco leaves, especially for Pb and Cu heavy metals.

Soil metal concentration is another key factor that influences heavy metal accumulation in plants. Higher soil metal concentrations generally lead to higher heavy metal uptake by plants, although this can vary depending on other soil properties, such as pH and organic matter content. In this paper, the content of Pb, Cd, and Cu in soil and tobacco showed a positive correlation, which also explained this problem. A great deal of work has been done in this field to illustrate this problem. For example, Duan et al. collected 107 soil and tobacco leaf samples in South China and clarified the quantitative relationship between soil properties and heavy metal content in tobacco leaves [45]. The results not only showed a positive correlation between the two but also provided a factual basis for the regression models based on soil factors to predict the contents of Pb, Cd, and Cu in tobacco in different regions.

### 5.2. Risk and Prevention of Heavy Metals

The risk assessment and prevention of heavy metals in agroecosystems have long been a focal point of attention [46]. This is because agricultural fields are complex systems influenced by various factors, including spatial variations in soil heavy metal content, physicochemical properties, climatic conditions, altitude, soil types, and farming practices [47, 48]. Among these factors, soil plays a crucial role in impacting economically important crops such as tobacco and can serve as a potential entry point for addressing the risks and prevention of heavy metals.

For common heavy metals, the enrichment coefficients of heavy metals in tobacco leaves were Cd > Hg > Pb > Cr > As, indicating that tobacco leaves can easily accumulate Cd [48]. Tobacco is known for its tendency to accumulate Cd, with an enrichment coefficient ranging from 5 to 10. In mature tobacco plants, Cd is usually found in leaves or stems, followed by roots and seeds. Tobacco products use leaves, so Cd control is the most important work in tobacco leaf production. Mei et al. also emphasized that Cd is a highly toxic heavy metal without any known biological function in plants, making it one of the most hazardous substances released into the environment [49]. Crops, including rice, maize, wheat, and tobacco, serve as significant sources of Cd exposure for humans. Cd toxicity adversely affects crop growth and development by disrupting crucial physiological and biochemical processes, ultimately posing a risk to human health throughout the food chain. Our study found that the Cd content in tobacco leaves was relatively high, with 4.89 mg/kg on average. Combined with the soil Cd excess rate of 12.1, it is not difficult to explain this problem. For the prevention and control of Cd heavy metals in tobacco leaves in the Longyan area, the eradication of Cd heavy metals in soil is an outstanding problem that needs to be solved urgently. In fact, in order to solve the problem of excessive Cd in tobacco leaves, previous studies have made many beneficial attempts, but the work mainly focused on soil improvement. For example, Zhang et al. proposed that low concentrations of NaCl could improve tolerance to Cd by reducing Cd accumulation and increasing antioxidant enzyme activity in tobacco [50]. Zeng et al. experimentally confirmed that the application of CaCO_3_ can significantly reduce the Cd accumulation in tobacco roots, stems, upper leaves, middle leaves, and lower leaves [51]. Zeng et al. further pointed out that the combined application of zeolite and silicon can significantly reduce the available Cd content in soil and the Cd content in tobacco [52]. Hu et al. believed that the application of nano-zeolite could improve soil properties, reducing available Cd content in soil and Cd content in tobacco [53]. Wang et al. discussed the impact of biofertilizers on alleviating Cd-induced growth inhibition of tobacco leaves [54]. Erdem pointed out that biochar from agricultural wastes can effectively reduce the mobility, absorption, and toxicity of Cd in plants [55]. However, according to Liu et al., it is believed that tobacco leaves have the capacity to accumulate approximately 80% of the total Cd extracted from the soil by the plant [56]. Despite the constraints in Cd-extraction observed in prominent tobacco cultivars, the screening of germplasm resources to identify varieties with high or low levels of Cd accumulation remains a crucial objective for future endeavors. Therefore, for the prevention and control of heavy metal Cd hazards in Longyan region's tobacco leaves, two approaches can be pursued. On the one hand, efforts should be made to enhance soil improvement, which may involve the addition of substances such as low concentrations of sodium chloride, nano-zeolite, CaCO_3_, biofertilizers, and biochar. On the other hand, it is necessary to breed tobacco varieties that are more tolerant to Cd elements.

In addition, the excessive presence of heavy metals such as Pb and Cu in tobacco is also a secondary concern that requires attention. Elevated levels of Pb and Cu in the soil can have the following impacts on tobacco plants: (1) Restricted plant growth: high concentrations of heavy metals can inhibit the growth and development of tobacco plants. They may impede root growth, cause leaf yellowing and shedding, and affect plant yield and quality. (2) Toxic effects of heavy metals at high concentrations: they can disrupt plant physiological processes such as photosynthesis, nutrient absorption, and water balance. This can lead to leaf damage, wilting, and death. (3) Accumulation of heavy metals in tobacco leaves can pose a threat to the health of smokers. When tobacco is burned, heavy metals can be released into the smoke and enter the human body through smoking. Long-term inhalation of smoke containing high concentrations of heavy metals can result in poisoning and health issues, such as central nervous system damage, cardiovascular diseases, and kidney damage, among others. Therefore, excessive levels of Pb and Cu in the soil have detrimental effects on tobacco plant growth and quality while further increasing the risk of smokers' exposure to these heavy metals. To mitigate the risk of heavy metal exceedance in tobacco, soil remediation measures and control of heavy metal pollution can be implemented. Previous studies have addressed this issue. Wang et al. conducted research to assess the impact of two organic fertilizers (OSiFA and OSiFB) and one mineral silicon fertilizer (MSiF) on tobacco plant Pb accumulation [57]. The results indicated that Si fertilizer reduced the availability of Pb in the soil by 28.6% and decreased Pb content in plant tissues by 17.2–25.6%, leading to an increase in plant biomass by 13.0–30.5%. Both organic and mineral silicon fertilizers can alleviate plant physiological stress and enhance tobacco growth by favorably influencing the soil microbial community's tolerance to heavy metals. Rong et al. discovered that, in comparison to untreated soil, the application of humic acid resulted in significant retention of available Pb and Cu in the soil, leading to reduced uptake of these metals in the upper, middle, and lower leaves of tobacco plants [58]. According to Cheng et al., it was observed that the application of tobacco stalk biochar resulted in increased soil nutrient levels (including nitrogen, phosphorus, and potassium contents), enhanced bacterial diversity indexes and richness, and altered the composition of the bacterial community [59]. These combined effects potentially contributed to the reduction in the mobility and bioavailability of both Pb and Cu in calcareous soil. Based on our findings, the excessive levels of heavy metals Pb and Cu in the soil of this region were relatively low, with rates of 0.951% and 1.12%, respectively. While this issue may not be significant in this particular area, it still warrants our attention.

In conclusion, the most significant issue of heavy metal contamination in the research area is primarily associated with Cd, followed by Cu and Pb. To mitigate the health risks associated with elevated levels of heavy metals, we have proposed models for heavy metals in tobacco leaves based on soil influencing factors. These models not only accurately predict the status of Pb, Cd, and Cu heavy metals in tobacco leaves in the region but also provide valuable insights for ensuring the safe production of tobacco in the study area.

## 6. Conclusion

The average levels of Pb, Cd, and Cu in the study area were found to be below the secondary standards of China and Fujian Province, with the percentages of samples exceeding the standards being 0.951%, 12.1%, and 1.12% for Pb, Cd, and Cu, respectively. The enrichment coefficients of Pb and Cd were calculated to be 19.3 and 54.3, respectively, while the enrichment coefficient of Cu was much lower at 1.07. Correlation and stepwise regression analysis revealed that the main soil factors affecting the content of Pb, Cd, and Cu in tobacco leaves were Pb, Cd, Cu, organic matter content, soil pH, and nutrients including K, N, and P. The soil pH was found to be negatively correlated with Pb, while K and P nutrients were negatively correlated with Cu content, and organic matter showed a positive correlation with Cd content in the tobacco leaves. The predominant concern regarding heavy metal contamination in the research area is primarily linked to Cd, followed by Cu and Pb. Implementing soil remediation measures or cultivating tobacco varieties that are tolerant to Cd, Cu, and Pb remains an ideal and effective approach for heavy metal prevention and control. Our prediction models for heavy metals in tobacco leaves have reached a significant level, providing a scientific basis for using soil property indexes in the sampling area to predict the risk of heavy metal pollution in tobacco leaves.

It is important to note that, besides soil characteristics, factors such as plant species, root structure, growth stage, temperature, humidity, and other environmental conditions also play a role in the accumulation of Pb, Cd, and Cu in plants. This study considered only those factors that had a significant impact on soil physical and chemical properties, and there is still potential for improvement in the model's correlation coefficient.

## Figures and Tables

**Table 1:** Physical and chemical properties of soils in tobacco-grown areas.

Item	Sample number	Min	Max	Average	Standard deviation	Coefficient of variation (%)
pH	421	3.37	8.64	5.81	0.471	8.1
Organic matter (g/kg)	421	6.70	205	29.1	15.9	54.6
Alkali-hydrolyzable nitrogen (mg/kg)	421	25.0	211	99.5	24.8	25.1
Available phosphorus (mg/kg)	421	5.07	159	37.4	16.5	44.2
Available potassium (mg/kg)	421	1.35	251	26.1	20.6	79.1
EC (mS/cm)	421	0.153	1.32	0.587	0.166	28.3
TOC (g/kg)	421	7.98	210	31.5	12.9	40.9

**Table 2:** Descriptive statistics of total and bioavailable concentrations of Pb, Cd, and Cu in soils.

Item	Sample number	Min (mg/kg)	Max (mg/kg)	Average (mg/kg)	Standard deviation	Coefficient of variation (%)	Secondary standard	Overlimit ratio (%)
Pb	421	1.39	49.9	12.1	6.99	57.9	28.0	0.951
Cd	421	0.003	0.950	0.092	0.063	68.3	0.150	12.1
Cu	421	0.031	19.1	3.88	2.41	62.1	15.0	1.12

**Table 3:** Heavy metal content and enrichment factors of Pb, Cd, and Cu in tobacco leaves.

ltem	Sample number	Min (mg/kg)	Max (mg/kg)	Average (mg/kg)	Standard deviation	Coefficient of variation (%)	Enrichment coefficient
Pb	421	0.430	7.08	2.33	0.765	32.9	19.3
Cd	421	0.221	13.2	4.89	1.90	38.8	54.3
Cu	421	1.23	9.15	4.35	1.16	26.6	1.07

**Table 4:** Correlation between heavy metals and other items in tobacco and soils.

		Soil								Tobacco		
		pH	N	P	K	OM	Pb	Cd	Cu	Pb	Cd	Cu
Soil	pH	1.00	−0.075	−0.187^*∗∗*^	−0.058	−0.123	−0.187^*∗∗*^	−0.025	0.044	−0.301^*∗∗*^	−0.031	0.079
	N	−0.075	1.00	−0.063	0.022	0.164^*∗∗*^	0.168^*∗∗*^	0.032	0.121	0.331^*∗∗*^	0.058	0.090
	P	−0.180^*∗∗*^	−0.063	1.00	0.202^*∗∗*^	−0.022	0.020	0.094	−0.203^*∗∗*^	0.094	0.086	−0.538^*∗∗*^
	K	−0.058	0.022	0.202^*∗∗*^	1.00	−0.021	0.031	0.035	−0.163^*∗∗*^	0.164^*∗∗*^	0.077	−0.289^*∗∗*^
	OM	−0.123^*∗∗*^	0.164^*∗∗*^	−0.022	−0.021	1.00	0.091	0.075	0.100	0.087^*∗∗*^	0.161^*∗∗*^	0.040
	Pb	−0.187^*∗∗*^	0.168^*∗∗*^	0.020	0.031	0.091	1.00	0.008	0.154^*∗∗*^	0.671^*∗∗*^	0.023	0.044
	Cd	−0.031	0.032^*∗∗*^	0.094	0.035^*∗∗*^	0.075	0.008	1.00	−0.200^*∗∗*^	0.030	0.877^*∗∗*^	−0.093
	Cu	−0.044	0.121	−0.203^*∗∗*^	−0.163^*∗∗*^	0.099	0.154^*∗∗*^	−0.200^*∗∗*^	1.00	0.032	−0.182^*∗∗*^	0.614^*∗∗*^

Tobacco	Pb	−0.301^*∗∗*^	0.331^*∗∗*^	0.085	0.164^*∗∗*^	0.087	0.671^*∗∗*^	0.030	0.033	1.00	0.052	−0.033
	Cd	−0.025	0.058	0.086	0.077	0.161^*∗∗*^	0.023	0.877^*∗∗*^	−0.182^*∗∗*^	0.052	1.00	−0.060
	Cu	0.079	0.090	−0.538^*∗∗*^	−0.289^*∗∗*^	0.044	0.040	−0.093	0.614^*∗∗*^	−0.033	−0.060	1.00

*Note*. ^*∗∗*^significant at 0.01 level; OM stands for organic matter.

**Table 5:** Prediction models of Pb, Cu, and Cd in tobacco leaves.

Item	Multiple regression equation	*R*	*R* ^2^	*P*
Pb	*Y*_Pb_=2.33 − 0.005*∗X*_K_+0.007*∗X*_N_ − 0.271*∗X*_pH_+0.065*∗X*_Pb_	0.802	0.787	<0.001
Cd	*Y*_Cd_=1.55+0.012*∗X*_OM_ − 0.014*∗X*_Cu_+34.6*∗X*_Cd_	0.901	0.891	<0.001
Cu	*Y*_Cu_=4.64 − 0.029*∗X*_P_ − 0.007*∗X*_K_+0.245*∗X*_Cu_	0.733	0.724	<0.001

*Note.* K: available potassium; N: alkali-hydrolyzable nitrogen; P: available phosphorus; OM: organic matter; *X*_Pb_, *X*_Cd_, and *X*_Cu_ represent the metal content of Pb, Cd, and Cu in soil; in the prediction model of Cd, *X*_Cu_ denotes the metal concentration of Cu in soil.

## Data Availability

The data that support the findings of this study are available from the corresponding author, Ping, upon reasonable request.
